# Cold Plasma as a Promising Non-Thermal Strategy for Enhancing Food Safety: A Review of Microbial and Mycotoxin Decontamination

**DOI:** 10.3390/molecules31030517

**Published:** 2026-02-02

**Authors:** Jiangqi Huang, Chenguang Zhou, Huiling Huang, Zhen Yang, Siyao Liu

**Affiliations:** 1 School of Food and Biological Engineering, Jiangsu University, Zhenjiang 212013, China; 2 School of Pharmacy, Jiangsu University, Zhenjiang 212013, China; 3 Key Laboratory of Nuclear Agricultural Sciences of Ministry of Agriculture and Zhejiang Province, Institute of Nuclear Agricultural Sciences, Zhejiang University, Hangzhou 310058, China

**Keywords:** cold plasma, non-thermal processing, food safety, microbial inactivation, mycotoxin degradation

## Abstract

Cold plasma (CP) is a novel non-thermal food processing technology characterized by low processing temperatures, high efficiency, and a pollution-free nature. It demonstrates promising application potential in food sterilization, preservation, and the degradation of mycotoxins. This review provides a comprehensive overview of recent advancements in the application of CP for food sterilization and mycotoxin degradation. It also critically analyzes the underlying degradation mechanisms of CP and the key factors affecting its decontamination efficacy. The application efficacy of CP across various food matrices is summarized, demonstrating its significant potential to reduce microbial loads and degrade major mycotoxins with minimal impact on food quality. Critical factors influencing treatment efficiency, particularly the matrix effect and process parameters, are analyzed. Furthermore, the review assesses the toxicological safety of the degradation products, citing evidence of reduced cytotoxicity in in vitro and in vivo models. It also discusses the major obstacles to industrial implementation, including limited penetration depth, challenges in equipment scale-up, and regulatory constraints. On this basis, the review outlines future research priorities, with particular emphasis on the development of intelligent control systems and the establishment of robust regulatory frameworks to support commercial application.

## 1. Introduction

Agricultural products are susceptible to contamination by fungi and mycotoxins throughout their entire lifecycle—from growth, harvest, and storage to processing and consumption. Toxigenic fungi, such as *Aspergillus*, *Fusarium*, and *Penicillium* species, grow under suitable temperature and humidity conditions and produce toxic secondary metabolites—mycotoxins [1,2,3]. These toxins contaminate approximately 25% of agricultural products globally [4], posing serious threats to human and animal health [5] and causing significant economic losses [6]. Consequently, the effective mitigation of fungal and mycotoxin contamination in food is crucial for both food safety and public health [7,8,9]. Common mycotoxins, including aflatoxins (Afs) [10,11], deoxynivalenol (DON) [12,13], zearalenone (ZEN) [14,15,16], fumonisins (FBs) [17,18,19], and ochratoxins (OTs) [20,21,22] exhibit potent hepatotoxicity, nephrotoxicity, neurotoxicity, carcinogenicity, teratogenicity, and mutagenicity [23,24]. For instance, AFs are classified as Group I carcinogens by the International Agency for Research on Cancer (IARC), being closely associated with liver cancer development [25,26].

To control these hazards, the food industry has long relied on conventional thermal, chemical, and physical methods [27,28]. Thermal treatments, such as pasteurization and sterilization, effectively inactivate microorganisms but degrade heat-sensitive nutrients (e.g., vitamins and functional proteins) and impair sensory qualities like color and flavor [29,30,31,32]. Furthermore, their efficacy is limited against thermally stable mycotoxins, AFs and DON, compromising product safety [4]. Chemical methods (e.g., ozone, ammoniation) can degrade toxins but risk chemical residues, environmental impact, and altered food flavor [29,30,33]. Physical adsorption often requires large adsorbent quantities, exhibits selective efficacy, and poses a desorption risk [31,32]. While biological methods offer specificity and mild operation, their narrow action spectrum is inadequate for multi-mycotoxin contamination, and they are often too costly and slow for industrial scale [34]. Other emerging non-thermal technologies like pulsed electric fields are also under investigation, yet they often face challenges regarding equipment complexity or limited efficacy against deeply embedded toxins. Therefore, developing a novel, non-thermal technology that synergistically ensures high efficiency, safety, cost-effectiveness, and minimal impact on food quality is a critical challenge.

Cold plasma, an ionized gas composed of reactive species, electrons, ions, and photons, has emerged as a promising technique [35,36,37,38,39]. Its key advantage lies in operating near ambient temperature (typically below 60 °C), thus preserving thermosensitive components and sensory properties of food [40,41]. CP efficiently generates ROS/RNS under atmospheric pressure, offering an environmentally friendly, residue-free processing method with short treatment times [42,43]. This review systematically elaborates on the core mechanisms of cold plasma in food sterilization and mycotoxin degradation, and analyzes its efficacy across different conditions. Building upon a discussion of its key influencing factors, special emphasis is placed on the critical impact of the matrix effect on treatment outcomes. It also includes a dedicated assessment of the toxicological safety of the resulting degradation products. Furthermore, this review summarized the attempts to bridge laboratory findings with scalable industrial applications, aiming to provide an in-depth discussion for research in this field.

## 2. Core Mechanisms of Cold Plasma Action

CP is a partially ionized gas often generated at atmospheric pressure. It contains a complex mixture of reactive species, including electrons, ions, radicals, and photons. Because the gas maintains a near-ambient temperature, it is highly effective for heat-sensitive food applications [44]. Various electrical discharge methods can produce CP, each with distinct characteristics suitable for different processing needs. The most prevalent configurations in food research include: Dielectric Barrier Discharge (DBD), Atmospheric Pressure Plasma Jet (APPJ), Corona Discharge (CD), Radio Frequency (RF) or Microwave (MW) Plasmas. Atmospheric Cold Plasma (ACP) is a broad term encompassing systems like DBD and APPJ operating at ambient pressure. The choice of configuration significantly influences the composition and flux of active species, thereby impacting the treatment efficacy and its suitability for specific food matrices and contamination scenarios [44]. The sterilization and detoxification efficacy of cold plasma is primarily attributed to the interaction between physical and chemical species produced during discharge [41,45]. Together, these active components inactivate microorganisms and degrade toxins by targeting multiple molecular sites simultaneously.

### 2.1. Microbial Inactivation Mechanisms

#### 2.1.1. Chemical Damage by Reactive Species

The basis of CP sterilization is its “cocktail” of chemically reactive species, primarily comprising reactive oxygen species (ROS) and reactive nitrogen species (RNS). Key ROS include ozone (O_3_), hydroxyl radicals (•OH), singlet oxygen (^1^O_2_), superoxide anion (O_2_•^−^), and atomic oxygen (O); significant RNS include nitric oxide (•NO) and nitrogen dioxide (•NO_2_), among others [46,47,48]. These reactive species induce irreversible damage to microbial cell structures. Firstly, they attack unsaturated fatty acids in the cell membrane, initiating a chain reaction of lipid peroxidation. This disrupts the structural integrity and fluidity of the membrane, leading to leakage of cellular contents [48,49]. Secondly, ROS/RNS can penetrate the compromised cell membrane, enter the cytoplasm, and oxidize or modify critical functional groups in proteins, such as thiol and amino groups. This leads to protein cross-linking, aggregation, or peptide chain scission, causing conformational changes and inactivation, ultimately inhibiting key enzymatic activities and normal cellular metabolism [50,51]. Research on *Aspergillus chevalieri* indicates that CP-induced oxidative stress initiates a cascade of critical intracellular disruptions, including membrane depolarization, calcium influx, mitochondrial dysfunction, and severe DNA damage [48,49,50,52,53]. This multi-pronged stress exceeds the capacity of the fungus’s protective responses (e.g., trehalose and chitin accumulation), resulting in rapid cell death.

#### 2.1.2. Physical Synergistic Effects

In addition to chemical damage, physical effects generated by CP contribute significantly to microbial inactivation [54]. Ultraviolet (UV) photons, particularly short-wave ultraviolet-C (UV-C) radiation emitted during plasma discharge, can be absorbed by microbial DNA, inducing the formation of pyrimidine dimers. This specifically inhibits DNA replication, contributing to the lethal effect [46,49]. Concurrently, the intense electric field present in the plasma can induce a transmembrane potential difference across the microbial cell membrane. When this potential exceeds a critical threshold, it triggers “electroporation,” creating transient or permanent pores in the membrane that drastically increase its permeability and accelerate the leakage of intracellular components [49]. Furthermore, the continuous “bombardment” of the cell surface by high-energy electrons and ions exerts a physical etching effect, directly eroding and disrupting the structural integrity of the cell wall and membrane [49].

#### 2.1.3. Synergistic Effects and Intracellular Dysregulation

Importantly, the interplay between these physical and chemical factors goes beyond a simple additive effect, involving complex spatiotemporal interactions that amplify their efficacy [55,56]. Initial physical bombardment and UV radiation weaken the cell’s physical barriers. The electroporation effect subsequently creates rapid pathways for reactive species like ROS/RNS to penetrate deep into the cell interior. Meanwhile, the specific DNA damage caused by UV radiation combines with the generalized oxidative damage inflicted by ROS/RNS. This effectively overwhelms cellular repair pathways, resulting in rapid and effective inactivation [4,49]. Recent studies suggest that reactive nitrogen species might act as signaling molecules interfering with microbial quorum sensing systems. Simultaneously, intense oxidative stress can exhaust intracellular antioxidant reserves, driving a metabolic collapse that parallels programmed cell death. These observations provide critical insights into the sterilization process from a systems biology perspective [49].

**Figure 1:**
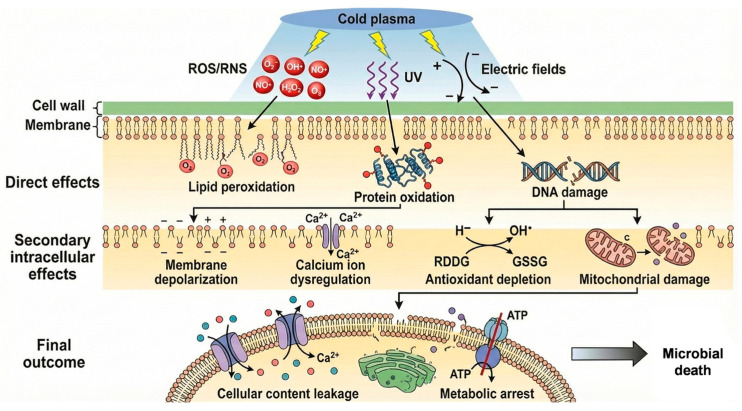
Proposed mechanisms of inactivation of microbes by cold plasma [52].

Following the initial structural damage, CP triggers a series of secondary intracellular disruptions that collectively drive microbial inactivation. Electric fields and oxidative species act together to deregulate calcium ion flow and depolarize the cell membrane, precipitating a breakdown in cellular homeostasis and signaling networks. Prolonged exposure to oxidative species drains the cell’s reservoir of reducing agents, particularly glutathione. This exhaustion of antioxidant defenses precipitates a fatal metabolic collapse. In fungal cells, this stress interferes with cell wall biosynthesis regulation, resulting in aberrant chitin deposition and compromised structural integrity. Concurrently, damage to the mitochondrial membrane and the electron transport chain induces a severe bioenergetic failure. By simultaneously targeting cell envelopes, genetic material, and metabolic pathways, CP creates a lethal environment that prevents recovery. This comprehensive damage profile highlights the unique, synergistic advantage of plasma technology [52]. Proposed mechanisms of inactivation of microbes by cold plasma is presented in Figure 1. Recent research has emphasized the “hurdle technology” approach by combining CP with other antimicrobial agents to amplify efficacy. For instance, integrated in-package treatments using hydrogen peroxide and atmospheric dielectric barrier discharge have shown synergistic inactivation of *E. coli* O157:H7 and *L. monocytogenes* on cabbage slices, achieving significantly higher reductions than individual treatments without altering food texture [57]. Similarly, the sequential application of 75% ethanol followed by CP has been found to significantly enhance microbial inactivation in complex matrices like bee pollen while simultaneously mitigating plasma-induced lipid oxidation through moisture control [58]. 

### 2.2. Mycotoxin Degradation Mechanisms

Unlike microbial inactivation, the core objective in degrading mycotoxins is to disrupt their stable chemical structures, particularly the key functional groups or bonds responsible for their toxicity.

#### 2.2.1. Attack by Reactive Species and Chemical Bond Cleavage

ROS and RNS generated by CP serve as the primary agents for mycotoxin degradation. These highly reactive particles form a hierarchical oxidation system with varying reactivities and lifetimes. Short-lived radicals like •OH, possessing extremely high reactivity, attack toxin molecules rapidly in situ. In contrast, longer-lived species such as O_3_ and hydrogen peroxide (H_2_O_2_) can diffuse into crevices and pores within the treatment matrix, enabling remote and sustained oxidation [59]. These reactive species selectively target vulnerable sites and “toxicogenic moieties” in the mycotoxin molecular structure, such as unsaturated double bonds, epoxy groups, and lactone rings [4,60]. Reactions such as electrophilic addition and oxidative cleavage compromise the structural integrity of the toxin. This leads to the fragmentation of the parent toxin into lower-molecular-weight byproducts with significantly reduced biological activity [31,60].

#### 2.2.2. Analysis of Degradation Pathways for Key Mycotoxins

The precise elucidation of mycotoxin degradation pathways has been achieved through advanced characterization using high-resolution mass spectrometry (HRMS) [61,62] and nuclear magnetic resonance (NMR) [61,62]. These studies reveal that CP treatment involves both the selective cleavage of toxic functional groups and a parallel series of complex reactions.

**Figure 2:**
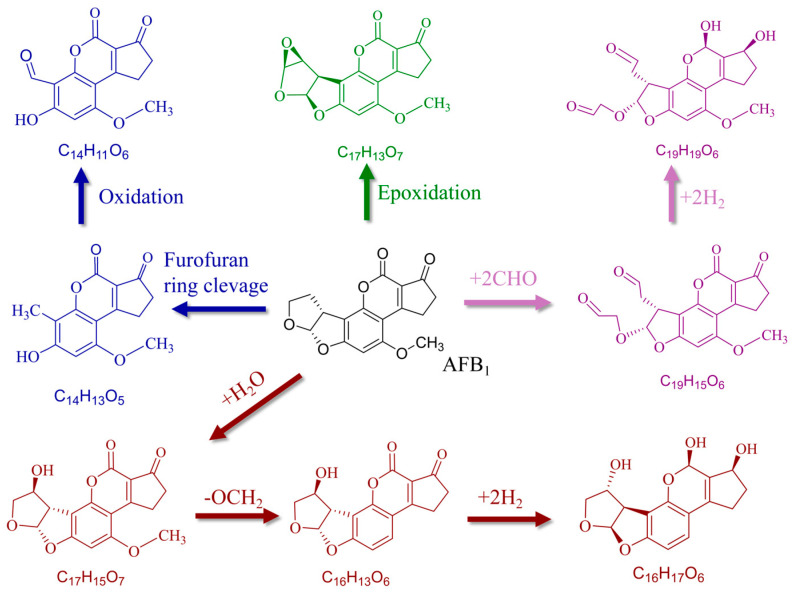
Possible degradation pathways of AFB1 in foods under high voltage DBD treatment, adapted from [46].

Reactive species (ROS/RNS) drive the degradation of Aflatoxin B1 (AFB1) by specifically attacking the C8=C9 double bond in the terminal furan ring, the structural feature responsible for its toxicity. The possible degradation pathways of AFB1 in foods under high voltage DBD treatment is presented in Figure 2. Studies indicate that AFB1 degradation consistently initiates with the rapid cleavage of this double bond [63]. For instance, Hojnik et al. [63] comprehensively analyzed degradation products using HRMS and NMR, confirming that degradation starts with electrophilic addition of water at the C8 position, followed by multiple oxidative pathways leading to the eventual ring-opening and degradation of the furan ring. The MS/MS fragmentation pattern can be delineated into three key steps: initial double cleavage at the carbonyl group resulting in the loss of a methyl group (generating fragments at m/z 285 and 257), followed by successive losses of carbonyl and methane, culminating in the complete destruction of the furan ring [63]. Similarly, Zhang Yan et al. [64]. inferred via HPLC-MS/MS that AFB1 degradation involves decomposition ring-opening and photo-elimination of the coumarin lactone ring, producing unstable intermediates that eventually polymerize, with key evidence being the identification of degradation products with molecular masses of 156, 298, and 338 Da. All these reactions result in the disappearance of the C8=C9 double bond, with this site being substituted by groups like hydroxyl or aldehyde, substantially reducing its carcinogenicity [46,63].

**Figure 3:**
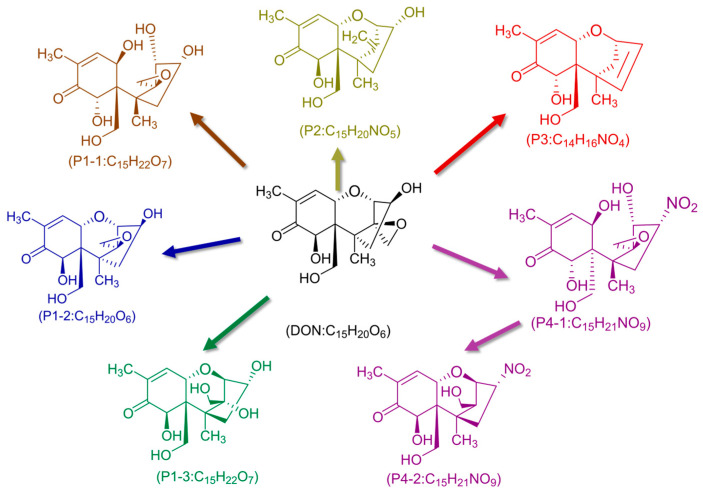
Possible pathways for the degradation of DON in foodstuffs under DBD treatment [47].

CP degradation of DON primarily focuses on its C12,13-epoxy group and the C9=C10 double bond [4]. Pathways involve direct oxidation by ROS and hydrolysis of the epoxy ring potentially facilitated by CP-induced environmental acidification. Chen et al. [47] elucidated the degradation mechanism of DON using mass spectrometry, revealing a complex reaction network. The plasma treatment primarily targets the C9=C10 double bond for addition reactions, opens the epoxy ring through deoxygenation or dihydroxylation, and substitutes hydroxyl groups with nitro moieties. These modifications collectively yield a diverse range of degradation derivatives. The possible pathways for the degradation of DON in foodstuffs under DBD treatment is presented in Figure 3. First-order kinetic studies also revealed that toxins with aliphatic chains (like DON) generally degrade faster than those with compact aromatic ring structures, reflecting differences in the stability of their chemical architectures [48].

The degradation of zearalenone (ZEN) targets the macrocyclic lactone structure and the C=C double bond on the phenolic ring, thereby eliminating its estrogenic activity [46]. The degradation likely proceeds via one of two primary pathways: the Criegee ozonolysis mechanism on the C=C bond, or direct bond cleavage driven by high-energy electron impact. In either scenario, subsequent oxidative addition by ROS yields characteristic aldehyde derivatives [46]. Wielogorska et al. [65] found that DBD treatment of ZEN generated three main degradation products, including isomers mono-hydroxylated at the C5 and C7 positions, and a product resulting from the opening of the lactone ring, confirming the successful disruption of the toxin’s core structure.

## 3. Application Efficacy of Cold Plasma Technology

Cold plasma technology has demonstrated excellent efficacy in microbial inactivation and mycotoxin degradation across various food matrices. To systematically assess its practical application potential, this section provides a comprehensive analysis of its performance in different food categories.

### 3.1. Application in Cereals and Their Products

Cereals are highly susceptible to mycotoxin contamination, particularly by deoxynivalenol (DON) and zearalenone (ZEN), posing a significant threat to food safety [66,67,68]. Research shows that atmospheric pressure cold plasma (ACP) treatment serves as an effective strategy for controlling such contamination. For instance, one study reported that ACP treatment of barley grains for 6 min reduced the DON concentration by 48.9% [4]. Another study on contaminated wheat demonstrated that ACP treatment for 15 min achieved a DON degradation rate of 61% [47]. In rice grain, CP treatment (25 kV) has demonstrated significant potential for controlling both molds and mycotoxins, with maximal reductions in deoxynivalenol (DON) and ochratoxin A (OTA) reaching 61.25% and 55.64%, respectively [69]. While major nutrients typically remain stable, CP can specifically affect certain protein components like prolamine and increase malondialdehyde (MDA) content, reflecting a degree of surface oxidation. Regarding ZEN degradation, notable efficacy has been observed; for example, in maize samples, ACP treatment at 50 kV for 120 s resulted in a ZEN degradation rate of 56.57% [70]. These degradation effects are primarily attributed to the abundant reactive oxygen and nitrogen species (e.g., ozone, hydroxyl radicals) generated during plasma processing, which can disrupt the molecular structure of mycotoxins [59].

Beyond effective toxin degradation, the impact of CP treatment on the quality of the cereals themselves is of considerable interest. Most studies suggest that under optimized conditions, CP treatment typically does not exert significant adverse effects on key quality parameters of grains. For example, a study on whole oat grains found that after treatment with dielectric barrier discharge (DBD) plasma under specific parameters (e.g., 37 W, 6 min), although aflatoxin B1 (AFB1) concentration was significantly reduced (82%), indicators such as protein content and pH showed no significant difference compared to the untreated control group [71]. Furthermore, research has confirmed that using low-pressure cold plasma to treat cereal seeds like wheat, oats, barley, and maize for 15 min achieved approximately a 3.0 log CFU/g reduction in *Aspergillus* fungi while successfully preserving seed germination vitality [72]. This demonstrates the biocompatibility and application potential of CP technology for surface decontamination and toxin degradation in cereals. In summary, ACP treatment shows significant efficacy in degrading DON, ZEN, and aflatoxins in cereals, generally without affecting their germination rate or major nutritional components (e.g., protein, β-glucan), indicating promising application prospects.

### 3.2. Application in Nuts and Oilseeds

Nuts such as peanuts, pistachios, and hazelnuts, as well as oilseeds like rapeseed, are high-risk commodities for contamination by aflatoxins (AFs) and zearalenone (ZEN) [73,74]. Studies show that CP technology is highly efficient in degrading mycotoxins in these products. For example, in the treatment of hazelnuts, using a DBD system for 12 min resulted in a reduction of over 70% for both total aflatoxins and AFB1 [75]. For peanuts, research indicates that DBD treatment for 10 min under 80% relative humidity air combined with post-treatment storage can nearly completely inactivate Aspergillus flavus spores without adversely affecting peanut quality attributes like color and texture; regarding toxin degradation, a 2 min treatment achieved a 71.3% reduction rate for AFB1 [76]. Another study reported that using 120 W radio-frequency plasma to treat peanuts for 150 s led to a 95% degradation rate for AFB1 [77]. These treatment efficacies are closely related to plasma type, working gas (especially humidity), treatment time, and power parameters [76,78].

In addition to efficient toxin degradation, the preservation of the sensory and physicochemical qualities of nuts post-CP treatment is crucial for assessing its suitability. Results from multiple sensory evaluation studies are encouraging. For instance, pistachios and hazelnuts treated with plasma jet received satisfactory scores for color, odor, taste, and overall acceptability [79,80]. Regarding quality metrics, studies report that for plasma jet-treated pistachios, indicators such as moisture content, peroxide value, free fatty acids, and total polyphenols showed no significant difference compared to untreated samples [79,81]. For oilseeds, such as rapeseed, research confirms that DBD-ACP treatment for 3 min can achieve a ZEN degradation rate of 91.6% [82]. These results highlight the significant potential of CP technology for post-harvest fungal inactivation, toxin degradation, and quality preservation in nuts and oilseeds, positioning it as a promising non-thermal processing technology.

### 3.3. Application in Spices

Dried spices are a challenging category, often having high initial microbial loads and mycotoxin risk [83,84]. Research has focused on microbial inactivation, with excellent results for some products. For instance, a specific study on saffron demonstrated that cold plasma treatment at 40 W for 10 min under an argon atmosphere reduced the total microbial load by 0.9 log cycles while maintaining the spice’s color, taste, and aroma with minimal loss [85]. Application of low-pressure cold plasma on various dried red peppers effectively reduced microbial contamination, with the optimal treatment time being individually determined for each type to ensure quality preservation [86]. The CP treatment protocol must be validated for each specific matrix, balancing decontamination against the preservation of its unique pigment and aroma profile [87]. Recent studies have expanded the application of CP to both dried and fresh spices. Pin-to-plate atmospheric cold plasma (ACP) has achieved complete decontamination of yeast and mold in cinnamon, black pepper, and fennel within 15 min at 230 V, while actually increasing the total phenolic content due to surface etching [88]. For fresh herbs such as rosemary, mint, and basil, 3-min ACP treatments can reduce natural microflora by up to 2.91 log CFU/g and extend shelf-life by 3–8 days [89,90].

### 3.4. Applications in Other Food Matrices

CP technology is being progressively explored for a wider range of food applications [91,92,93,94]. For instance, using plasma-activated water (PAW) for washing fresh fruits and vegetables can effectively reduce surface microbial loads and pesticide residues [95,96,97]. In sundried tomatoes, Surface Dielectric Barrier Discharge (SDBD) cold plasma treatment significantly reduced naturally occurring fungal spores (e.g., *Aspergillus* spp.), with efficacy dependent on fungal species, while concurrently preserving product pH and water activity and even enhancing the extractability of bioactive compounds like lycopene [98]. Furthermore, applications have been extended to dairy matrices, where CP has been studied for microbial control on liquid milk, powder, and cheese surfaces, alongside investigations into its potential effects on enzyme activity [99,100,101,102,103]. In the category of meat, poultry, and eggs, research demonstrates CP’s ability to inactivate pathogens on fresh meat and poultry surfaces, as well as to disinfect eggshells, thereby contributing to extended shelf life [104,105,106]. Additionally, in-package cold plasma (ICP) technology offers a novel approach for extending the shelf life of perishable foods like meat and seafood products, as it enables direct sterilization within sealed packages, effectively minimizing the risk of post-processing contamination [42,49]. CP technology also shows promise for mycotoxin decontamination in liquid foods. In jujube juice, a brief cold plasma treatment significantly reduced Alternaria mycotoxins (AOH and AME) in a voltage-dependent manner. Importantly, this effective decontamination was achieved without compromising the juice’s fundamental quality, underscoring its value as a green and non-thermal processing alternative [107]. CP is also proving effective for low-water activity powders. In soybean powder, low-pressure CP (0.35 mbar) using O_2_ as the working gas achieved over a 5-log reduction in *B. cereus* within 25 min, significantly outperforming synthetic air treatments due to the high concentration of oxygen-derived radicals [108]. To provide a clearer overview of CP application outcomes, Table 1 summarizes specific data from various studies on its sterilization and mycotoxin degradation efficacy in different food matrices.

## 4. Key Factors Influencing Plasma Treatment Efficacy

**Figure 4:**
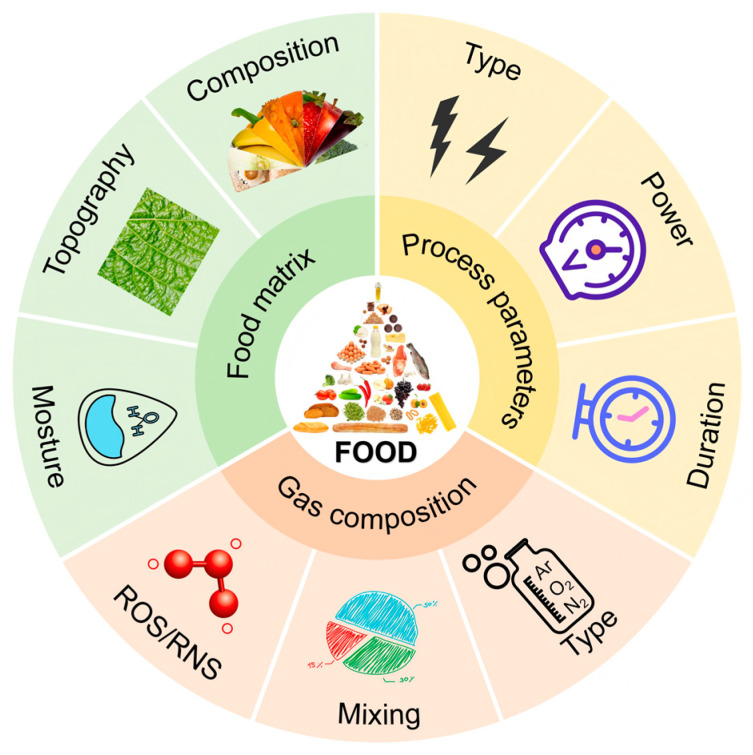
Factors exerting a crucial influence on the efficacy of plasma treatment.

The efficacy of cold plasma treatment is variable and depends on a complex interplay of factors related to equipment design, process parameters, working gas composition, and food matrix properties (as shown in Figure 4). A deep understanding of these factors and their mechanisms is essential for process optimization and targeted application.

### 4.1. Influence of Process Parameters

The type of plasma generator such as DBD, APPJ, RF plasma, as well as other configurations like Surface DBD (SDBD) and CD, each with distinct discharge characteristics and application suitability. Their specific operational parameters are primary determinants of treatment outcome [133]. Generally, increasing the discharge voltage/power or extending the treatment duration leads to the generation of higher concentrations and a wider variety of reactive species, thereby enhancing both sterilization and detoxification effects [46,47,70]. For example, the degradation rate of DON under dry conditions increased significantly with ACP treatment time, from 25.1% at 5 min to 75.9% at 60 min [4]. However, these parameters cannot be increased indefinitely, as excessively intense treatment may adversely affect food quality. Therefore, finding the optimal balance between efficacy and quality preservation is necessary [134].

### 4.2. Influence of Working Gas

The type and composition of the working gas directly dictate the types and relative abundances of ROS and RNS produced in the plasma, profoundly influencing the treatment outcome [135]. Using air as the working gas is cost-effective and produces a broad spectrum of both ROS and RNS, conferring wide-ranging antimicrobial and detoxification capabilities. This makes it highly attractive for potential industrial-scale applications [48]. Gases like argon (Ar) or helium (He) facilitate the generation of more stable and uniform plasma discharges due to their lower ionization energies. However, as they are not direct sources of reactive species, they are often used in combination with small admixtures of reactive gases to tailor the reactive species output [48]. Introducing small amounts of oxygen (O_2_) into an inert gas background is a common strategy to enhance the production of strongly oxidative ROS (e.g., atomic oxygen O, ozone O_3_), significantly boosting the oxidative degradation of microbes and toxins [48,134]. Nitrogen gas (N_2_) is primarily used to promote the formation of RNS. By carefully adjusting the mixing ratios of different gases, it is possible to selectively modulate the reactive species composition to suit specific treatment objectives [48].

### 4.3. Influence of Food Matrix Properties

The inherent physical and chemical properties of the food matrix itself represent the most complex yet critical influence on CP treatment efficacy, collectively known as the “matrix effect.”

Moisture content is a crucial factor [136,137,138]. The presence of water serves as a precursor for generating highly reactive •OH radicals via photolysis or electron-impact dissociation during plasma discharge, which can dramatically enhance degradation efficiency [4,5]. The findings demonstrate a clear disparity in degradation efficiency. In solution, DON was entirely degraded (100%) within 5 min, whereas the dry state yielded only 75.9% degradation after a 60 min treatment [4]. This stark contrast highlights a major challenge in transitioning CP technology from laboratory studies on pure compounds to real-world food applications.

In addition, the surface topography of food (e.g., roughness, crevices, porosity) creates a “shielding effect” [139,140]. Microorganisms and toxin molecules located within recesses or pores are shielded from direct exposure to plasma-generated active species, creating “dead zones” that can significantly reduce the overall treatment effectiveness [49].

Furthermore, the intrinsic biochemical composition of the food, including the presence of antioxidants (e.g., ascorbic acid, polyphenols), proteins, lipids, and carbohydrates, can scavenge plasma-generated reactive species, thereby quenching the treatment efficacy [141,142,143]. This ‘chemical quenching’ effect means that the functional groups and redox potential of the food matrix itself constitute a critical, and often variable, determinant of CP treatment outcome [46,63]. Consequently, a dry, rough-surfaced, and lipid-rich nut presents a significantly greater challenge for CP treatment compared to a smooth, moist fruit surface, due to the combined limitations of lacking the aqueous medium for •OH generation, physical shielding, and chemical quenching. Understanding these factors as an integrated “matrix effect” is vital for developing strategies to overcome these hurdles, such as designing treatments with improved penetration or combining CP with surface wetting agents. To better understand the complex parameters affecting CP efficacy, researchers have begun employing machine learning and meta-analysis. 19A large-scale analysis of inactivation kinetics identified “dissipated power per plasma volume” (W/cm^3^) as the most critical integrated predictor of decontamination efficacy, alongside the matrix category and microbial genus [144].

## 5. Food Quality and Safety Assessment

### 5.1. Impact on Food Nutritional and Sensory Quality

A major advantage of CP as a non-thermal technology is its potential to largely preserve the original nutritional and sensory attributes of food. However, the highly reactive species generated can also induce some unintended chemical modifications.

Multiple studies report that under optimized treatment conditions, CP has minimal impact on the overall quality of various foods [4,31,49]. Nonetheless, specific changes have been observed depending on conditions. For instance, treatments using air plasma can lead to the formation of nitrous and nitric acids from RNS reactions with water, potentially causing a slight decrease in the surface pH of the treated food [145].

Lipid oxidation is a key concern when applying CP to high-fat foods. ROS can initiate the oxidation of unsaturated fatty acids, potentially leading to the development of off-flavors and a reduction in shelf life [146,147,148,149]. Proteins may also undergo oxidation, resulting in modifications of amino acid side chains or alterations in protein structure, which can affect their functional properties [150,151,152,153]. The impact on sensitive components like vitamins and pigments is contingent upon the specific treatment intensity and the food matrix [49,60,154,155,156,157]. Overall, by precise control of process parameters, it is feasible to achieve effective sterilization and detoxification while minimizing negative impacts on food quality.

### 5.2. Toxicological Safety Assessment of Degradation Products

Establishing the safety of degradation products is paramount for the regulatory approval and consumer acceptance of cold plasma technology. It is critical to recognize that degradation does not automatically guarantee complete detoxification, as primary products may undergo further reactions such as nitration or oxidation. Therefore, a systematic evaluation of the complete degradation pathway and the toxicity of the resulting products is essential [48].

Encouragingly, existing evidence from both in vitro and in vivo studies indicates that CP degradation effectively reduces mycotoxin toxicity. In vitro studies using cell models, including human hepatoma HepG2 and colon carcinoma Caco-2 cells, have consistently demonstrated that the cytotoxicity of degradation products from CP-treated AFB_1_ and DON is markedly lower than that of the parent toxins [46,47]. For instance, one study reported that ACP treatment of a DON solution for 20 min significantly reduced its cytotoxicity, resulting in over 80% viability of Caco-2 cells [46]. This reduction in toxicity is corroborated by in vivo studies. Bioassays using Artemia larvae and investigations in rat models have shown that the median lethal dose (LD_50_) of CP-treated AFB_1_ degradation products is substantially higher than that of untreated AFB_1_, indicating a clear reduction in acute toxicity [46].

The fundamental basis for this observed detoxification lies in the CP-induced destruction of key chemical structures essential for the toxins’ biological activity, such as the difuran ring in AFB_1_ or the epoxy group in DON, which impairs their ability to interact with critical cellular targets [46].

In summary, current research, spanning chemical structural analysis to biological evaluation, indicates substantially reduced toxicity of CP degradation products compared to the parent toxins. Nonetheless, while existing assessments generally show no increase in cytotoxicity, they remain insufficient to fully confirm the absolute safety of these products within complex food systems [48]. Future, more comprehensive studies are warranted, including validation in additional cell lines, more extensive in vivo toxicological investigations, and a particular focus on potential mutagenicity, to ultimately ensure the safe application of CP technology in food.

## 6. Challenges for Industrial Application and Future Prospects

### 6.1. Current Technical and Economic Challenges

Despite promising laboratory-scale results, the widespread industrial adoption of cold plasma technology faces several significant hurdles. Firstly, scalability and uniformity present a major challenge, as most research has been conducted using small-scale, batch-style laboratory reactors, while designing industrial-scale systems capable of continuous, uniform, and efficient treatment of large volumes of irregularly shaped food commodities (e.g., bulk grains) requires substantial engineering development [5,47,158]. Furthermore, CP is inherently limited by its penetration depth, functioning primarily as a surface or near-surface technology due to the limited ability of its active species to penetrate solid matrices, which is a significant constraint for scenarios where contaminants like mycotoxins have infiltrated the interior of seeds or grains [134,146]. Another critical consideration is cost and energy consumption; although using air as a process gas is inexpensive, the initial capital investment for industrial-grade plasma equipment can be high, and the operational energy consumption remains a pivotal factor in determining its economic feasibility compared to existing technologies [46,47]. Finally, regulatory approval constitutes a major hurdle, as CP must undergo rigorous safety assessment by bodies such as the US FDA and EFSA in Europe, a process that demands comprehensive data on the safety of the treatment process itself, the toxicological profile of any degradation products, and the long-term effects on the nutritional quality of treated foods [49,134]. From a technology readiness level perspective, cold plasma technology for food processing is currently transitioning from laboratory validation to the development of industrial prototypes. A central challenge in this phase is the dynamic optimization of process parameters (e.g., power, gas composition, exposure time) for diverse food matrices with varying geometries, surface textures, and compositions. Consequently, the development of “intelligent” plasma systems incorporating real-time monitoring sensors and adaptive feedback control mechanisms is crucial for achieving consistent, uniform, and effective treatment of complex food products, representing a key bridge linking scientific proof-of-concept to viable industrial application.

### 6.2. Future Key Research Directions

To advance the practical implementation of cold plasma technology, future research efforts should concentrate on several key areas. First, a deeper mechanistic understanding is required, necessitating further fundamental research to elucidate the detailed chemical kinetics of interactions between plasma-generated reactive species and various food components (e.g., proteins, lipids, carbohydrates). This knowledge is crucial for better predicting and controlling treatment outcomes and potential side reactions [46,49]. Second, process optimization and the development of hybrid technologies are essential; this involves not only the systematic optimization of parameters for specific food-mycotoxin combinations but also exploring “hurdle” approaches where CP is integrated with other mild technologies (e.g., mild heating, UV, ultrasound) [159]. Such synergies hold the promise of overcoming inherent limitations like penetration depth and achieving superior results with lower overall energy input [4,49]. Concurrently, the design and engineering of industrial equipment represent a critical path forward. Interdisciplinary collaboration is vital to develop cost-effective, reliable, and automated plasma systems that can be integrated into existing food production lines for continuous processing [46,49]. Finally, comprehensive studies on safety and quality impacts are imperative. Long-term, systematic evaluations must thoroughly assess the effects of CP processing on the nutritional value, sensory attributes, shelf-life stability, and the long-term toxicological profile of degradation products across a wide range of foods. These studies will provide the robust scientific data necessary for securing regulatory approvals and building consumer confidence [46,47].

## 7. Conclusions

This review substantiates CP as a viable non-thermal strategy for the simultaneous sterilization of food surfaces and the degradation of major mycotoxins, including aflatoxins, deoxynivalenol, and zearalenone. The technology is driven by the combined action of reactive species, UV radiation, and electric fields, which effectively inactivate pathogens and cleave toxic functional groups. However, bridging the gap between laboratory success and industrial adoption remains a challenge. The efficiency of the process is heavily modulated by the food matrix itself: variations in moisture, surface roughness, and composition often lead to inconsistent treatment, hindering the development of scalable, continuous-processing systems. To advance commercialization, equipment design must evolve to ensure consistent treatment across complex food matrices, and safety claims must be validated through comprehensive toxicological studies. Furthermore, integrating cold plasma with other non-thermal methods offers a practical route to improve energy efficiency. Solving these engineering and safety constraints is the final step required to validate CP as a scalable, sustainable solution for the modern food industry.

## Figures and Tables

**Table 1:** Effect of cold plasma on sterilization and mycotoxin degradation in different food matrices.

Food Matrix	Target Contaminant	Plasma Type	Key Process Parameters	Effect	Reference
Cereals					
Barley	DON	DBD	Air, 6 min	Degradation Rate 48.9% on barley grains	[4]
Wheat	DON	DBD	50 kV, 8 min, 20% moisture	Degradation Rate 36.1%	[31]
Corn	ZEN	DBD	50 kV, 120 s	Degradation per-centage of the zearalenone in maize reach 56.57%	[70]
Corn	AFB_1_	HVACP	Air, 40% humidity, 5 min	AFB1 was degraded by 76%.	[46]
Oat Flour	T-2 & HT-2 toxins	Low-pressure DBD	N_2_ gas, 30 min	T-2 and HT-2 Degradation Rate 43.3% and 38.9%	[47]
Wheat	Yeast and mold	Negative CD	10 kV, 50 Hz; 15 min	Reduction of >1.95 and 1.6 log_10_ CFU/g	[109]
Wheat	*S. cerevisiae* *E. coli*	Plasma activated water	1 and 24 h	Significant reduction in *E. coli* after 1 h of Exposure; significant decrease in *S. cerevisiae* at 24 h	[110]
Wheat bran	Total Plate count	Plasma activated water	40 min	Total plate counts in bran were significantly decreased by 68.85%	[111]
Oats	Total plate count and fungal count	Low-temperature plasma treatment (LTPT)	0–20 min, 60 kV	Reduction of 1.84 and 1.35 log_10_ CFU/g	[112]
Nuts/Oilseeds					
Peanut	AFB_1_	RF	120 W, 150 s	Degradation Rate 95%	[77]
Hazelnut	AFs (B1, B2, G1, G2)	DBD	N_2_/O_2_ mixture, 12 min	The cold plasma resulted in loss of function and structure, and ultimately cell death of fungal cells; Degradation Rate >70%	[47,60]
Canola grain, canola meal and barley grains	ZEN	DBD	3 min	The ZEA degradation rates after 3 min DBD-ACP treatment on canola grain, canola meal, and barley grains were 91.6, 83.2, and 64.8%, respectively	[82]
Pistachio kernels	*A. flavus* AFs (B1, B2, G1, G2)	DBD	Argon/Air mixture, 10–20 kV,15 min	The highest fungal reduction of 5.14 log CFU/g. The maximal aflatoxin reduction obtained (B1, 83.70%; B2, 74.36%; G1, 41.39%; G2, 50.74%)	[81]
Mixed nuts	AFs (B1, B2, G1, G2)	APCP	Air,600 V, 0–16 min	Degradation Rate: total AF of 72.6%	[113]
Raw pistachio	*A. flavus*	DBD	Air, 10.7 kV, 18 min	Complete removal of *A. flavus* post-treatment	[114]
Peanuts	*A. flavus* and *A. niger*	Plasma jet	Argon, 200 W, 30 L/min, 3.5 min	Reduced to undetectable level	[115]
Peeled peanuts	*A. flavus*	Plasma jet	Ar/O_2_ mix: 9 L/min/90 mL/min, 20 W, 10 min	Achieved 100% microbial growth inhibition	[116]
Spices					
Black pepper-corns	*S.enteritidis* *B. tequilensis*	DBD	Helium, 10.3 kV, 22.1 min	Reduction 3.4 log CFU/g 1.7 log spores/g	[117]
Red pepper powder	*B. cereus*	Combination of MH and DBD	MH (60 °C for 5–20 min) and DBD plasma (5–20 min)	Reducing B. cereus counts ≥ 6.0 log CFU/g	[118]
Thyme and paprika	Total bacterial count	DBD	6 kHz, 5 min	Reduction 1.18 log cycle	[119]
Black pepper	*S. enterica*, *B. subtilis spores* and *B. atrophaeus*	RF	Argon, 30 W, 10 L/min, 15 min	*S. enterica*, *B. subtilis spores* and *B. atrophaeus* spores were reduced by 4.1, 2.4 and 2.8 log, respectively	[120]
Cinnamon, black pepper, fennel	Yeast and mold count	ACP	230 V, 15 min	Reduction 1.3 Log CFU/g, 1.1 Log_10_ CFU/g, 1.0 Log CFU/g	[88]
Onion	*S. enteritidis*, *E. coli O157:H7*, and *L. monocytogenes*	DBD	He; 15 kV; 20 min	3.1, 1.4, and 1.1 log_10_ CFU/cm^2^ reductions in achieved, respectively	[121]
Blank peppercorns	*B. tequilensis*	Simultaneous CP and UV treatment	10.3 kV; 22.1 min	1.7 log_10_ spores/g reduction in *B. tequilensis* and 3.4 log10 CFU/g in indigenous bacteria	[117]
Dried peppermint	*E. coli O157:H7*	Low pressure cold plasma	50 W and 60 W; 20 min	Significant reduction in *E. coli O157:H7*	[122]
Black pepper grains	Microbes	Cold plasma	Atmospheric air; 15 kV and 30 kV; 3–20 min	All microbes sustained a 2.92 log_10_ reduction after a 24 h period post-treatment	[123]
Others					
Beef	Total viable counts Fungi and yeast	PAW produced by APPJ	Air, 600 W, 1 min, plasma activated water	Efficiently inactivation of the total bacteria, fungi and yeast in beef by 0.83–1.76 logs	[124]
Chicken breast	*Salmonella enterica serovar*	DBD plasma	14.5 W, 10 min	*Salmonella* counts on chicken breast samples after 10 min of exposure were reduced by 3.7 Log_10_CFUs	[125]
Lamb meat	*Brochothrix thermosphacta*	DBD plasma	80 kV, 50 Hz, 5 min	ACP (80 kV) treatment inactivated *B. thermosphacta* in PBS within 30 s, while a 5 min treatment achieved only a 2 Log reduction in a complex meat medium	[126]
Fresh and frozen pork	*E. coli* *L. monocytogenes*	Corona discharge plasma jet	58 kHz, 20 kV, 90–120 s	Treatment of pork samples for 120 s reduced *E. coli* O157:H7 by 1.5 log and *L. monocytogenes* by >1.0 log	[127]
Beef jerky	*L. innocua*	Plasma beam system	N2 or air, 20 kHz, 300 W, brine (sodium nitrite) solution	Samples cured with air-plasma showed 0.5 log *L. innocua* inactivation in the brine and 0.85 log *L. innocua* inactivation in the jerky	[128]
Milk	*E. coli*, *L. monocytogenes*, *S. typhimurium*	Encapsulated DBD plasma	250 W; 15 kHz; ambient air; 5 and 10 min	Reduction 2.4 log_10_ CFU/mL for each	[129]
Cow’s milk	*S. aureus, E. coli*, and *L. monocytogenes*	DBD	Voltage: 40–80 V; Time: 15–120 s	Broken bacterial cell membrane; reduced activity of metabolic enzymes, and bacterial DNA destruction	[130]
Blueberry	Bacteria and fungi	DBD	36 V; 1.8 A; 0–10 min	Decrease in number of bacteria by 93.0% and fungi by 25.8% after a 10 min treatment	[131]
Carrots	Aerobic mesophiles, yeast and molds·	DBD	100 kV; 60 Hz; 5 min	About 2.1 log_10_ CFU/g reduction for total aerobic mesophiles, yeast and molds	[132]

## Data Availability

No new data were created or analyzed in this study.

## References

[1] Liu J., Zhang X., Tian J., Li Y., Liu Q., Chen X., Feng F., Yu X., Yang C. (2022). Multiomics Analysis Reveals That Peach Gum Colouring Reflects Plant Defense Responses against Pathogenic Fungi. Food Chem..

[2] Godana E.A., Yang Q., Zhang X., Zhao L., Wang K., Dhanasekaran S., Mehari T.G., Zhang H. (2023). Biotechnological and Biocontrol Approaches for Mitigating Postharvest Diseases Caused by Fungal Pathogens and Their Mycotoxins in Fruits: A Review. J. Agric. Food Chem..

[3] Zhang Y., Zhao C., Picchetti P., Zheng K., Zhang X., Wu Y., Shen Y., De Cola L., Shi J., Guo Z. (2024). Quantitative SERS Sensor for Mycotoxins with Extraction and Identification Function. Food Chem..

[4] Feizollahi E., Arshad M., Yadav B., Ullah A., Roopesh M.S. (2021). Degradation of Deoxynivalenol by Atmospheric-Pressure Cold Plasma and Sequential Treatments with Heat and UV Light. Food Eng. Rev..

[5] Feizollahi E., Iqdiam B., Vasanthan T., Thilakarathna M.S., Roopesh M.S. (2020). Effects of Atmospheric-Pressure Cold Plasma Treatment on Deoxynivalenol Degradation, Quality Parameters, and Germination of Barley Grains. Appl. Sci..

[6] Zhang H., Zhang B., He H., Zhang L., Hu X., Wu C. (2025). Fungicidal Effect of Strong Oxidative Free Radicals against Fusarium Graminearum and Their Impact on Wheat Growth and Yield. Agriculture.

[7] Dai C., Zhang L., Ma H., Yin X., He R., Qian J. (2017). Ultrasound-Assisted Detoxification of Free Gossypol from Cottonseed Meal. J. Food Process Eng..

[8] Gao S., Zhang Y., Sun Q., Guo Z., Zhang D., Zou X. (2024). Enzyme-Assisted Patulin Detoxification: Recent Applications and Perspectives. Trends Food Sci. Technol..

[9] Ngolong Ngea G.L., Yang Q., Castoria R., Zhang X., Routledge M.N., Zhang H. (2020). Recent Trends in Detecting, Controlling, and Detoxifying of Patulin Mycotoxin Using Biotechnology Methods. Compr. Rev. Food Sci. Food Saf..

[10] Mwakosya A.W., Limbu S.M., Majaliwa N., Zou X., Shi J., Kibazohi O. (2022). Aflatoxin B_1_ Variations in Animal Feeds along the Supply Chain in Tanzania and Its Possible Reduction by Heat Treatment. Food Agric. Immunol..

[11] Nasir U., Naeem I., Asif M., Ismail A., Gong Y.Y., Routledge M.N., Amjad A., Fazal A., Ismail Z. (2021). Assessment of Aflatoxins Exposure through Urinary Biomarker Approach and the Evaluation of the Impacts of Aflatoxins Exposure on the Selected Health Parameters of the Children of Multan City of Pakistan. Food Control.

[12] Qi Y., Yang Y., Hassane Hamadou A., Li B., Xu B. (2022). Gentle Debranning as a Technology to Reduce Microbial and Deoxynivalenol Levels in Common Wheat (*Triticum aestivum* L.) and Its Application in Milling Industry. J. Cereal Sci..

[13] Shen L., Wang Y., Li X., Hou Z., Mao J., Shi J., Battino M., Routledge M.N., Gong Y., Zou X. (2024). Spatial-Temporal Distribution of Deoxynivalenol, Aflatoxin B1, and Zearalenone in the Solid-State Fermentation Basin of Traditional Vinegar and Their Potential Correlation with Microorganisms. Food Chem..

[14] Guo Z., Gao L., Yin L., Arslan M., El-Seedi H.R., Zou X. (2023). Novel Mesoporous Silica Surface Loaded Gold Nanocomposites SERS Aptasensor for Sensitive Detection of Zearalenone. Food Chem..

[15] Luo L., Ma S., Li L., Liu X., Zhang J., Li X., Liu D., You T. (2019). Monitoring Zearalenone in Corn Flour Utilizing Novel Self-Enhanced Electrochemiluminescence Aptasensor Based on NGQDs-NH_2_-Ru@SiO_2_ Luminophore. Food Chem..

[16] Wang S., Niu R., Yang Y., Wang Y. (2020). Development and Evaluation of a Rapid Immunomagnetic Extraction for Effective Detection of Zearalenone in Agricultural Products. Food Control.

[17] Qiu J., Jiang C., Wang S., He C., Chen D., Lan J., Xu J., Lee Y.-W., Shi J. (2025). Geographic Variations in the *Fusarium* Species and Toxins Associated with Maize Ear Rot in China. Int. J. Food Microbiol..

[18] Qiu J., Gu H., Wang S., Ji F., He C., Jiang C., Shi J., Liu X., Shen G., Lee Y.-W. (2024). A Diverse *Fusarium* Community Is Responsible for Contamination of Rice with a Variety of *Fusarium* Toxins. Food Res. Int..

[19] Hu J., Lv H., Hou M., Wang G., Lee Y.-W., Shi J., Gu Z., Xu J. (2020). Preparative Isolation and Purification of B-Type Fumonisins by Using Macroporous Resin Column and High-Speed Countercurrent Chromatography. Food Addit. Contam. Chem. Anal. Control Expo. Risk Assess..

[20] Wei M., Dhanasekaran S., Yang Q., Ngolong Ngea G.L., Godana E.A., Zhang H. (2022). Degradation and Stress Response Mechanism of *Cryptococcus podzolicus* Y3 on Ochratoxin a at the Transcriptional Level. LWT.

[21] Shang L., Bai X., Chen C., Liu L., Li M., Xia X., Wang Y. (2019). Isolation and Identification of a *Bacillus megaterium* Strain with Ochratoxin a Removal Ability and Antifungal Activity. Food Control.

[22] Zhu W., Li L., Zhou Z., Yang X., Hao N., Guo Y., Wang K. (2020). A Colorimetric Biosensor for Simultaneous Ochratoxin a and Aflatoxins B1 Detection in Agricultural Products. Food Chem..

[23] Wang B., Wei W., Zhang Y., Xu H., Ma H. (2022). Decontamination and Quality Assessment of Freshly Squeezed Grape Juice under Spiral Continuous Flow-through Pulsed Light (SCFPL) Treatment. J. Food Process. Preserv..

[24] Mukunzi D., Habimana J.d.D., Li Z., Zou X. (2023). Mycotoxins Detection: View in the Lens of Molecularly Imprinted Polymer and Nanoparticles. Crit. Rev. Food Sci. Nutr..

[25] Xia X., Zhang Y., Li M., Garba B., Zhang Q., Wang Y., Zhang H., Li P. (2017). Isolation and Characterization of a *Bacillus subtilis* Strain with Aflatoxin B1 Biodegradation Capability. Food Control.

[26] Zhang D., Yang W., Li X., Zou X., Niu L., Gao S. (2025). Integrating Magnetic-Plasmonic and Membrane-like Nanotags for the Sensitive and Reliable Detection of Aflatoxin B1 in Foodstuffs. Food Control.

[27] Wang Y., Hu Z., Wang B., Liao J., Zhang M. (2022). Non-Thermal Microbial Inactivation of Honey Raspberry Wine through the Application of High-Voltage Electrospray Technology. Food Bioprocess Technol..

[28] Osae R., Essilfie G., Alolga R.N., Akaba S., Song X., Owusu-Ansah P., Zhou C. (2020). Application of Non-thermal Pretreatment Techniques on Agricultural Products Prior to Drying: A Review. J. Sci. Food Agric..

[29] Boateng I.D., Yang X.-M. (2021). Thermal and Non-Thermal Processing Affect Maillard Reaction Products, Flavor, and Phytochemical Profiles of *Ginkgo Biloba* Seed. Food Biosci..

[30] Boateng I.D., Zhang W., Li Y.-Y., Saalia F.K., Yang X.-M. (2022). Non-Thermal Pretreatment Affects Ginkgo Biloba L. Seed’s Product Qualities, Sensory, and Physicochemical Properties. J. Food Sci..

[31] Pankaj S.K., Shi H., Keener K.M. (2018). A Review of Novel Physical and Chemical Decontamination Technologies for Aflatoxin in Food. Trends Food Sci. Technol..

[32] Azam K., Akhtar S., Gong Y.Y., Routledge M.N., Ismail A., Oliveira C.A.F., Iqbal S.Z., Ali H. (2021). Evaluation of the Impact of Activated Carbon-Based Filtration System on the Concentration of Aflatoxins and Selected Heavy Metals in Roasted Coffee. Food Control.

[33] Villacrés-Granda I., Proaño A., Coello D., Debut A., Vizuete K., Ballesteros I., Granda-Albuja G., Rosero-Mayanquer H., Battino M., Giampieri F. (2021). Effect of Thermal Liquefaction on Quality, Chemical Composition and Antibiofilm Activity against Multiresistant Human Pathogens of Crystallized Eucalyptus Honey. Food Chem..

[34] Alexandre A.P.S., Castanha N., Calori-Domingues M.A., Augusto P.E.D. (2017). Ozonation of Whole Wheat Flour and Wet Milling Effluent: Degradation of Deoxynivalenol (DON) and Rheological Properties. J. Environ. Sci. Health B.

[35] Zhou C., Hu Y., Zhou Y., Yu H., Li B., Yang W., Zhai X., Wang X., Liu J., Wang J. (2024). Air and Argon Cold Plasma Effects on Lipolytic Enzymes Inactivation, Physicochemical Properties and Volatile Profiles of Lightly-Milled Rice. Food Chem..

[36] Zhou C., Zhou Y., Tang Q., Sun Y., Ji F., Wu J., Yu H., Liu T., Yang W., Liu S. (2024). Impact of Argon Dielectric Barrier Discharge Cold Plasma on the Physicochemical and Cooking Properties of Lightly-Milled Rice. Innov. Food Sci. Emerg. Technol..

[37] Lin L., Liao X., Cui H. (2019). Cold Plasma Treated Thyme Essential Oil/Silk Fibroin Nanofibers against *Salmonella* Typhimurium in Poultry Meat. Food Packag. Shelf Life.

[38] Yang X., Ma L., Zheng J., Qiao Y., Bai J., Cai J. (2024). Effects of Atmospheric Pressure Plasma Treatment on the Quality and Cellulose Modification of Brown Rice. Innov. Food Sci. Emerg. Technol..

[39] Konchekov E.M., Gusein-Zade N., Burmistrov D.E., Kolik L.V., Dorokhov A.S., Izmailov A.Y., Shokri B., Gudkov S.V. (2023). Advancements in Plasma Agriculture: A Review of Recent Studies. Int. J. Mol. Sci..

[40] Li B., Peng L., Cao Y., Liu S., Zhu Y., Dou J., Yang Z., Zhou C. (2024). Insights into Cold Plasma Treatment on the Cereal and Legume Proteins Modification: Principle, Mechanism, and Application. Foods.

[41] Hu Y., Zhu Y., Aalim H., Cao Y., Peng L., Dou J., Ma Y., Zhai X., Guo Z., Cai J. (2024). Cold Plasma-Assisted Pretreatment for Fabrication and Characterization of Rice Starch-Stearic Acid Complexes. Food Biosci..

[42] Zhang Y., Zhang J., Zhang Y., Hu H., Luo S., Zhang L., Zhou H., Li P. (2021). Effects of In-Package Atmospheric Cold Plasma Treatment on the Qualitative, Metabolic and Microbial Stability of Fresh-Cut Pears. J. Sci. Food Agric..

[43] Nasiru M.M., Frimpong E.B., Muhammad U., Qian J., Mustapha A.T., Yan W., Zhuang H., Zhang J. (2021). Dielectric Barrier Discharge Cold Atmospheric Plasma: Influence of Processing Parameters on Microbial Inactivation in Meat and Meat Products. Compr. Rev. Food Sci. Food Saf..

[44] Liu S., Yang D., Huang J., Huang H., Sun J., Yang Z., Zhou C. (2025). Advances in Atmospheric Cold Plasma Technology for Plant-Based Food Safety, Functionality, and Quality Implications. Foods.

[45] Shishir M.R.I., Kamal M.M., Karim N., Saifullah M., Khan S., Zhang K., Marappan G., Aalim H., Hashim S.B.H., Zhai X. (2025). Cold Plasma and Integrated Approaches for Fresh Produce Preservation: Mechanisms, Quality Attributes, Challenges, and Future Directions. Trends Food Sci. Technol..

[46] Shi H., Cooper B., Stroshine R.L., Ileleji K.E., Keener K.M. (2017). Structures of Degradation Products and Degradation Pathways of Aflatoxin B1 by High-Voltage Atmospheric Cold Plasma (HVACP) Treatment. J. Agric. Food Chem..

[47] Chen X., Qiu Y., Zhang J., Guo Y., Ding Y., Lyu F. (2022). Degradation Efficiency and Products of Deoxynivalenol Treated by Cold Plasma and Its Application in Wheat. Food Control.

[48] Wielogorska E., Ahmed Y., Meneely J., Graham W.G., Elliott C.T., Gilmore B.F. (2019). A Holistic Study to Understand the Detoxification of Mycotoxins in Maize and Impact on Its Molecular Integrity Using Cold Atmospheric Plasma Treatment. Food Chem..

[49] Ott L.C., Appleton H.J., Shi H., Keener K., Mellata M. (2021). High Voltage Atmospheric Cold Plasma Treatment Inactivates *Aspergillus Flavus* Spores and Deoxynivalenol Toxin. Food Microbiol..

[50] Shen C., Chen W., Li C., Cui H., Lin L. (2023). The Effects of Cold Plasma (CP) Treatment on the Inactivation of Yam Peroxidase and Characteristics of Yam Slices. J. Food Eng..

[51] Wei W., Yang S., Yang F., Hu X., Wang Y., Guo W., Yang B., Xiao X., Zhu L. (2023). Cold Plasma Controls Nitrite Hazards by Modulating Microbial Communities in Pickled Radish. Foods.

[52] Molina-Hernandez J.B., Capelli F., Laurita R., Tappi S., Laika J., Gioia L., Valbonetti L., Chaves-Lopez C. (2022). A Comparative Study on the Antifungal Efficacy of Cold Atmospheric Plasma at Low and High Surface Density on *Aspergillus chevalieri* and Mechanisms of Action. Innov. Food Sci. Emerg. Technol..

[53] Bai J.-W., Li D.-D., Abulaiti R., Wang M., Wu X., Feng Z., Zhu Y., Cai J. (2025). Cold Plasma as a Novel Pretreatment to Improve the Drying Kinetics and Quality of Green Peas. Foods.

[54] Pan J., Zhang Z., Mintah B.K., Xu H., Dabbour M., Cheng Y., Dai C., He R., Ma H. (2022). Effects of Nonthermal Physical Processing Technologies on Functional, Structural Properties and Digestibility of Food Protein: A Review. J. Food Process Eng..

[55] Zhu Y., Li C., Cui H., Lin L. (2020). Feasibility of Cold Plasma for the Control of Biofilms in Food Industry. Trends Food Sci. Technol..

[56] Lin L., Liao X., Li C., Abdel-Samie M.A., Cui H. (2020). Inhibitory Effect of Cold Nitrogen Plasma on *Salmonella* Typhimurium Biofilm and Its Application on Poultry Egg Preservation. LWT.

[57] Kim Y.E., Myung G.E., Jeon Y.J., Min S.C. (2024). Integrated In-Package Treatment of Hydrogen Peroxide and Cold Plasma for Microbial Inactivation of Cabbage Slices. Food Sci. Biotechnol..

[58] Chen M., Yang X., Ji Z., Zhao H., Cheng N., Cao W. (2024). Combined Treatment of Drying, Ethanol, and Cold Plasma for Bee Pollen: Effects on Microbial Inactivation and Quality Attributes. Food Biosci..

[59] Shi H., Ileleji K., Stroshine R.L., Keener K., Jensen J.L. (2017). Reduction of Aflatoxin in Corn by High Voltage Atmospheric Cold Plasma. Food Bioprocess Technol..

[60] Misra N.N., Yadav B., Roopesh M.S., Jo C. (2019). Cold Plasma for Effective Fungal and Mycotoxin Control in Foods: Mechanisms, Inactivation Effects, and Applications. Compr. Rev. Food Sci. Food Saf..

[61] Mei S., Chen X. (2023). Investigation into the Anti-Inflammatory Mechanism of Coffee Leaf Extract in LPS-Induced Caco-2/U937 Co-Culture Model through Cytokines and NMR-Based Untargeted Metabolomics Analyses. Food Chem..

[62] Zhang W., Jiang X., Liu L., Zhao Y., Bai F., Wang J., Gao R., Xu X. (2024). The Influence Mechanism of Phospholipids Structure and Composition Changes Caused by Oxidation on the Formation of Flavor Substances in Sturgeon Caviar. Food Chem..

[63] Hojnik N., Modic M., Walsh J.L., Žigon D., Javornik U., Plavec J., Žegura B., Filipič M., Cvelbar U. (2021). Unravelling the Pathways of Air Plasma Induced Aflatoxin B1 Degradation and Detoxification. J. Hazard. Mater..

[64] Zhang Y., Wang A., Xiao J., Wang S., Jiang W., Li Y. (2015). Effect and Pathway Analysis of Aflatoxins B1 in Degradation of Acetonitrile-Dissolved by Low Temperature Radio Frequency Plasma. J. Chin. Cereals Oils Assoc..

[65] Ten Bosch L., Pfohl K., Avramidis G., Wieneke S., Viöl W., Karlovsky P. (2017). Plasma-Based Degradation of Mycotoxins Produced by Fusarium, Aspergillus and Alternaria Species. Toxins.

[66] Ma S., Wang M., You T., Wang K. (2019). Using Magnetic Multiwalled Carbon Nanotubes as Modified QuEChERS Adsorbent for Simultaneous Determination of Multiple Mycotoxins in Grains by UPLC-MS/MS. J. Agric. Food Chem..

[67] Luo L., Liu X., Ma S., Li L., You T. (2020). Quantification of Zearalenone in Mildewing Cereal Crops Using an Innovative Photoelectrochemical Aptamer Sensing Strategy Based on ZnO-NGQDs Composites. Food Chem..

[68] He P., Mehedi Hassan M., Yang W., Shi Z., Zhou X., Xu Y., Ouyang Q., Chen Q. (2023). Rapid and Stable Detection of Three Main Mycotoxins in Rice Using SERS Optimized AgNPs@K30 Coupled Multivariate Calibration. Food Chem..

[69] Guo J., He Z., Ma C., Li W., Wang J., Lin F., Liu X., Li L. (2023). Evaluation of Cold Plasma for Decontamination of Molds and Mycotoxins in Rice Grain. Food Chem..

[70] Zheng Z., Niu L., Yang W., Chen Y., Huang Y., Li C. (2023). Degradation of Zearalenone by Dielectric Barrier Discharge Cold Plasma and Its Effect on Maize Quality. Foods.

[71] Dousti M., Bashiry M., Zohrabi P., Siahpoush V., Ghaani A., Abdolmaleki K. (2024). The Effect of Dielectric Barrier Discharge (DBD) Cold Plasma Treatment on the Reduction of Aflatoxin B1 and the Physicochemical Properties of Oat. Appl. Food Res..

[72] Selcuk M., Oksuz L., Basaran P. (2008). Decontamination of Grains and Legumes Infected with *Aspergillus* spp. and *Penicillum* spp. by Cold Plasma Treatment. Bioresour. Technol..

[73] Tang C., He Y., Yuan B., Li L., Luo L., You T. (2024). Simultaneous Detection of Multiple Mycotoxins in Agricultural Products: Recent Advances in Optical and Electrochemical Sensing Methods. Compr. Rev. Food Sci. Food Saf..

[74] Xu L., Chen Z., Bai X., Deng J., Zhao X., Jiang H. (2025). Determination of Aflatoxin B1 in Peanuts Based on Millimetre Wave. Food Chem..

[75] Siciliano I., Spadaro D., Prelle A., Vallauri D., Cavallero M.C., Garibaldi A., Gullino M.L. (2016). Use of Cold Atmospheric Plasma to Detoxify Hazelnuts from Aflatoxins. Toxins.

[76] Tang L., Cao W., Keener K.M. (2024). Effect of Dielectric Barrier Discharge High Voltage Atmospheric Cold Plasma on *Aspergillus flavus* Inactivation and Aflatoxin B1 Degradation on Inoculated Raw Peanuts. Innov. Food Sci. Emerg. Technol..

[77] Wang S.-Q., Huang G.-Q., Li Y.-P., Xiao J.-X., Zhang Y., Jiang W.-L. (2015). Degradation of Aflatoxin B1 by Low-Temperature Radio Frequency Plasma and Degradation Product Elucidation. Eur. Food Res. Technol..

[78] Mošovská S., Medvecká V., Gregová M., Tomeková J., Valík Ľ., Mikulajová A., Zahoranová A. (2019). Plasma Inactivation of *Aspergillus Flavus* on Hazelnut Surface in a Diffuse Barrier Discharge Using Different Working Gases. Food Control.

[79] Makari M., Hojjati M., Shahbazi S., Askari H. (2021). Elimination of Aspergillus Flavus from Pistachio Nuts with Dielectric Barrier Discharge (DBD) Cold Plasma and Its Impacts on Biochemical Indices. J. Food Qual..

[80] Sen Y., Onal-Ulusoy B., Mutlu M. (2019). *Aspergillus* Decontamination in Hazelnuts: Evaluation of Atmospheric and Low-Pressure Plasma Technology. Innov. Food Sci. Emerg. Technol..

[81] Esmaeili Z., Hosseinzadeh Samani B., Nazari F., Rostami S., Nemati A. (2023). The Green Technology of Cold Plasma Jet on the Inactivation of *Aspergillus Flavus* and the Total Aflatoxin Level in Pistachio and Its Quality Properties. J. Food Process Eng..

[82] Feizollahi E., Roopesh M.S. (2021). Degradation of Zearalenone by Atmospheric Cold Plasma: Effect of Selected Process and Product Factors. Food Bioprocess Technol..

[83] Zhai W., You T., Ouyang X., Wang M. (2021). Recent Progress in Mycotoxins Detection Based on Surface-Enhanced Raman Spectroscopy. Compr. Rev. Food Sci. Food Saf..

[84] Xia G., Li Y., Tao H., Zhang L., Zhang J., Yang H., Mustapha A.T., Zhou C. (2022). Inactivation Mechanism of Catalytic Infrared against *Pseudomonas Aeruginosa* and Its Decontamination Application on Dry Green Sichuan Pepper (*Zanthoxylum schinifolium*). Food Control.

[85] Birjandi Toroghi Z., Niazmand R., Moradinezhad F., Bayat H. (2024). Potential of Cold Plasma Pretreatment for Preserving Biochemical Attributes and Ensuring the Microbiological Safety of Saffron Stigma. Food Sci. Nutr..

[86] Dikmetas D.N., Zargarchi S., Scharf S., Gökoglu B.Z.-, Capanoglu E., Karbancioglu-Güler F., Gök R., Esatbeyoglu T. (2025). Low Pressure Cold Plasma Treatment for Microbial Decontamination, Improvement of Bioaccessibility and Aroma Profile of Dried Red Peppers. Food Chem..

[87] Gavahian M., Cullen P.J. (2020). Cold Plasma as an Emerging Technique for Mycotoxin-Free Food: Efficacy, Mechanisms, and Trends. Food Rev. Int..

[88] Kahar S.P., Shelar A., Annapure U.S. (2024). Effect of Pin-to-Plate Atmospheric Cold Plasma (ACP) on Microbial Load and Physicochemical Properties in Cinnamon, Black Pepper, and Fennel. Food Res. Int..

[89] Özdemir E., Başaran P., Kartal S., Akan T. (2025). Effect of Non-Thermal Atmospheric Cold Plasma on Surface Microbial Inactivation and Quality Properties of Fresh Herbs and Spices. Foods.

[90] Zhang J., Du Q., Yang Y., Zhang J., Han R., Wang J. (2023). Research Progress and Future Trends of Low Temperature Plasma Application in Food Industry: A Review. Molecules.

[91] Cui H., Li H., Abdel-Samie M.A., Surendhiran D., Lin L. (2021). Anti-*Listeria monocytogenes* Biofilm Mechanism of Cold Nitrogen Plasma. Innov. Food Sci. Emerg. Technol..

[92] Xu H., Dai C., Tang Y., Xu X., Umego E.C., He R., Ma H. (2022). The Selective Breeding and Mutagenesis Mechanism of High-Yielding Surfactin Bacillus Subtilis Strains with Atmospheric and Room Temperature Plasma. J. Sci. Food Agric..

[93] Shen C., Chen W., Aziz T., Khojah E., Al-Asmari F., Alamri A.S., Alhomrani M., Cui H., Lin L. (2024). Drying Kinetics and Moisture Migration Mechanism of Yam Slices by Cold Plasma Pretreatment Combined with Far-Infrared Drying. Innov. Food Sci. Emerg. Technol..

[94] Yu P., Zhu W., Qiao Y., Yang X., Ma L., Cai Y., Cai J. (2025). The Effect of Gliding Arc Discharge Low-Temperature Plasma Pretreatment on Blueberry Drying. Foods.

[95] Li M., Shi T., Wang X., Bao Y., Xiong Z., Monto A.R., Jin W., Yuan L., Gao R. (2022). Plasma-Activated Water Promoted the Aggregation of *Aristichthys Nobilis* Myofibrillar Protein and the Effects on Gelation Properties. Curr. Res. Food Sci..

[96] Li M., Wang X., Shi T., Xiong Z., Jin W., Bao Y., Monto A.R., Yuan L., Gao R. (2024). Mechanism of Plasma-Activated Water Promoting the Heat-Induced Aggregation of Myofibrillar Protein from Silver Carp (*Aristichthys Nobilis*). Innov. Food Sci. Emerg. Technol..

[97] Thirumdas R., Kothakota A., Annapure U., Siliveru K., Blundell R., Gatt R., Valdramidis V.P. (2018). Plasma Activated Water (PAW): Chemistry, Physico-Chemical Properties, Applications in Food and Agriculture. Trends Food Sci. Technol..

[98] Molina-Hernandez J.B., Laika J., Peralta-Ruiz Y., Palivala V.K., Tappi S., Cappelli F., Ricci A., Neri L., Chaves-López C. (2022). Influence of Atmospheric Cold Plasma Exposure on Naturally Present Fungal Spores and Physicochemical Characteristics of Sundried Tomatoes (*Solanum lycopersicum* L.). Foods.

[99] Nikmaram N., Keener K.M. (2022). The Effects of Cold Plasma Technology on Physical, Nutritional, and Sensory Properties of Milk and Milk Products. LWT.

[100] Rathod N.B., Kahar S.P., Ranveer R.C., Annapure U.S. (2021). Cold Plasma an Emerging Nonthermal Technology for Milk and Milk Products: A Review. Int. J. Dairy Technol..

[101] Koorehpaz K., Amiri S., Vardast M.R., Yousefi M. (2025). The Application of Cold Plasma on Microbial and Physicochemical Properties of Milk. J. Food Process Eng..

[102] Akarca G., Atik A., Atik İ., Denizkara A.J. (2023). The Use of Cold Plasma Technology in Solving the Mold Problem in Kashar Cheese. J. Food Sci. Technol..

[103] Fayaz U., Srivastava S., Manzoor S., Pandey V.K., Shams R., Dar A.H., Dash K.K., Hanan E. (2025). Microwave Powered Cold Plasma Applications for Food Quality and Safety: A Review. J. Food Process Eng..

[104] Csadek I., Vankat U., Schrei J., Graf M., Bauer S., Pilz B., Schwaiger K., Smulders F.J.M., Paulsen P. (2023). Treatment of Ready-to-Eat Cooked Meat Products with Cold Atmospheric Plasma to Inactivate Listeria and Escherichia Coli. Foods.

[105] Roh S.H., Oh Y.J., Lee S.Y., Kang J.H., Min S.C. (2020). Inactivation of Escherichia Coli O157:H7, Salmonella, Listeria Monocytogenes, and Tulane Virus in Processed Chicken Breast via Atmospheric in-Package Cold Plasma Treatment. LWT.

[106] Abdoli B., Khoshtaghaza M.H., Ghomi H., Torshizi M.A.K., Mehdizadeh S.A., Pishkar G., Dunn I.C. (2024). Cold Atmospheric Pressure Air Plasma Jet Disinfection of Table Eggs: Inactivation of Salmonella Enterica, Cuticle Integrity and Egg Quality. Int. J. Food Microbiol..

[107] Wang X., Han Y., Niu H., Zhang L., Xiang Q., Zong W. (2022). *Alternaria* Mycotoxin Degradation and Quality Evaluation of Jujube Juice by Cold Plasma Treatment. Food Control.

[108] Teresa Fernández-Felipe M., Inés Valdez-Narváez M., Martinez A., Rodrigo D. (2024). Oxygen and Air Cold Plasma for the Inactivation of *Bacillus Cereus* in Low-Water Activity Soy Powder. Food Res. Int..

[109] Naumenko N., Potoroko I., Velyamov M., Krasulya O., Dunchenko N. (2022). Application of Cold Plasma Radiation to Inactivate Toxigenic Molds and Reduce the Risk of Contamination of Wheat Grain. AIP Conf. Proc..

[110] Jirešová J., Scholtz V., Julák J., Šerá B. (2022). Comparison of the Effect of Plasma-Activated Water and Artificially Prepared Plasma-Activated Water on Wheat Grain Properties. Plants.

[111] Suo T., Guo X.-N., Zhu K.-X. (2022). Effects of Tempering with Plasma-Activated Water on Total Plate Count and Quality Properties of Wheat Flour. J. Cereal Sci..

[112] Liu S., He T., Rafique H., Zou L., Hu X. (2022). Effect of Low-Temperature Plasma Treatment on the Microbial Inactivation and Physicochemical Properties of the Oat Grai. Cereal Chem..

[113] Dinç M., Taşan M., Palabiyik I., Akbulut M.T., Gunes R. (2025). Effect of Atmospheric Pressure Cold Plasma on Aflatoxins in Mixed Nuts: A Comparative Study Using Natural and Spiked Samples. Food Chem..

[114] Dasan B.G., Mutlu M., Boyaci I.H. (2016). Decontamination of *Aspergillus flavus* and *Aspergillus parasiticus* Spores on Hazelnuts via Atmospheric Pressure Fluidized Bed Plasma Reactor. Int. J. Food Microbiol..

[115] Lin C.-M., Patel A.K., Chiu Y.-C., Hou C.-Y., Kuo C.-H., Dong C.-D., Chen H.-L. (2022). The Application of Novel Rotary Plasma Jets to Inhibit the Aflatoxin-Producing *Aspergillus flavus* and the Spoilage Fungus, *Aspergillus niger* on Peanuts. Innov. Food Sci. Emerg. Technol..

[116] Intanon W., Vichiansan N., Leksakul K., Boonyawan D., Kumla J., Suwannarach N., Lumyong S. (2021). Inhibition of the Aflatoxin-Producing Fungus *Aspergillus Flavus* by a Plasma Jet System. J. Food Process. Preserv..

[117] Bang I.H., Kim Y.E., Lee S.Y., Min S.C. (2020). Microbial Decontamination of Black Peppercorns by Simultaneous Treatment with Cold Plasma and Ultraviolet C. Innov. Food Sci. Emerg. Technol..

[118] Jeon E.B., Choi M.-S., Kim J.Y., Park S.Y. (2020). Synergistic Effects of Mild Heating and Dielectric Barrier Discharge Plasma on the Reduction of Bacillus Cereus in Red Pepper Powder. Foods.

[119] Rezaee Z. (2019). Application of Non-Thermal Plasma for Decontamination of Thyme and Paprika. Int. J. Food Allied Sci..

[120] Hertwig C., Reineke K., Ehlbeck J., Knorr D., Schlüter O. (2015). Decontamination of Whole Black Pepper Using Different Cold Atmospheric Pressure Plasma Applications. Food Control.

[121] Kim J.H., Min S.C. (2018). Moisture Vaporization-Combined Helium Dielectric Barrier Discharge-Cold Plasma Treatment for Microbial Decontamination of Onion Flakes. Food Control.

[122] Kashfi A.S., Ramezan Y., Khani M.R. (2020). Simultaneous Study of the Antioxidant Activity, Microbial Decontamination and Color of Dried Peppermint (*Mentha piperita* L.) Using Low Pressure Cold Plasma. LWT.

[123] Charoux C.M.G., Free L., Hinds L.M., Vijayaraghavan R.K., Daniels S., O’Donnell C.P., Tiwari B.K. (2020). Effect of Non-Thermal Plasma Technology on Microbial Inactivation and Total Phenolic Content of a Model Liquid Food System and Black Pepper Grains. LWT.

[124] Liao X., Xiang Q., Cullen P.J., Su Y., Chen S., Ye X., Liu D., Ding T. (2020). Plasma-Activated Water (PAW) and Slightly Acidic Electrolyzed Water (SAEW) as Beef Thawing Media for Enhancing Microbiological Safety. LWT.

[125] Aboubakr H.A., Nisar M., Nayak G., Nagaraja K.V., Collins J., Bruggeman P.J., Goyal S.M. (2020). Bactericidal Efficacy of a Two-Dimensional Array of Integrated, Coaxial, Microhollow, Dielectric Barrier Discharge Plasma against Salmonella Enterica Serovar Heidelberg. Foodborne Pathog. Dis..

[126] Patange A., Boehm D., Bueno-Ferrer C., Cullen P.J., Bourke P. (2017). Controlling *Brochothrix thermosphacta* as a Spoilage Risk Using in-Package Atmospheric Cold Plasma. Food Microbiol..

[127] Choi S., Puligundla P., Mok C. (2016). Corona Discharge Plasma Jet for Inactivation of Escherichia Coli O157:H7 and Listeria Monocytogenes on Inoculated Pork and Its Impact on Meat Quality Attributes. Ann. Microbiol..

[128] Inguglia E.S., Oliveira M., Burgess C.M., Kerry J.P., Tiwari B.K. (2020). Plasma-Activated Water as an Alternative Nitrite Source for the Curing of Beef Jerky: Influence on Quality and Inactivation of *Listeria innocua*. Innov. Food Sci. Emerg. Technol..

[129] Kim H.-J., Yong H.I., Park S., Kim K., Choe W., Jo C. (2015). Microbial Safety and Quality Attributes of Milk Following Treatment with Atmospheric Pressure Encapsulated Dielectric Barrier Discharge Plasma. Food Control.

[130] Wu X., Luo Y., Zhao F., M S.M., Mu G. (2021). Influence of Dielectric Barrier Discharge Cold Plasma on Physicochemical Property of Milk for Sterilization. Plasma Process. Polym..

[131] Dong X.Y., Yang Y.L. (2019). A Novel Approach to Enhance Blueberry Quality during Storage Using Cold Plasma at Atmospheric Air Pressure. Food Bioprocess Technol..

[132] Kumar Mahnot N., Siyu L.-P., Wan Z., Keener K.M., Misra N.N. (2020). In-Package Cold Plasma Decontamination of Fresh-Cut Carrots: Microbial and Quality Aspects. J. Phys. D Appl. Phys..

[133] Domonkos M., Tichá P., Trejbal J., Demo P. (2021). Applications of Cold Atmospheric Pressure Plasma Technology in Medicine, Agriculture and Food Industry. Appl. Sci..

[134] Feizollahi E., Misra N.N., Roopesh M.S. (2021). Factors Influencing the Antimicrobial Efficacy of Dielectric Barrier Discharge (DBD) Atmospheric Cold Plasma (ACP) in Food Processing Applications. Crit. Rev. Food Sci. Nutr..

[135] Lin L., Liao X., Li C., Abdel-Samie M.A., Siva S., Cui H. (2021). Cold Nitrogen Plasma Modified Cuminaldehyde/*β*-Cyclodextrin Inclusion Complex and Its Application in Vegetable Juices Preservation. Food Res. Int..

[136] Tang Z., Li Y., Zhang B., Wang M., Li Y. (2020). Controlling Rice Leaf Breaking Force by Temperature and Moisture Content to Reduce Breakage. Agronomy.

[137] Chandio F.A., Li Y., Ma Z., Ahmad F., Syed T.N., Shaikh S.A., Tunio M.H. (2021). Influences of Moisture Content and Compressive Loading Speed on the Mechanical Properties of Maize Grain Orientations. Int. J. Agric. Biol. Eng..

[138] Aalim H., Hashim S.B.H., Zhou C., Zou X., Luo Z. (2024). Matrix Characteristics Modulate Black Rice Phenolic Compounds Bioaccessibility and Antioxidant Activity during Simulated Gastrointestinal Digestion. Food Biosci..

[139] Zhang J., Yu X., Xu B., Yagoub A.E.A., Mustapha A.T., Zhou C. (2021). Effect of Intensive Pulsed Light on the Activity, Structure, Physico-Chemical Properties and Surface Topography of Polyphenol Oxidase from Mushroom. Innov. Food Sci. Emerg. Technol..

[140] Yang X., Ma L., Yu P., Qiao Y., Feng Z., Bai J., Zhou R., Wang C., Cai J. (2025). The Comparative Evaluation of the Quality of Brown Rice by Plasma Treatment and Milling Treatment: Appearance, Cooking Characteristics, Texture Characteristics, and Nutrient Composition. J. Cereal Sci..

[141] Chai Z., Tian L., Yu H., Zhang L., Zeng Q., Wu H., Yan Z., Li D., Hutabarat R.P., Huang W. (2020). Comparison on Chemical Compositions and Antioxidant Capacities of the Green, Oolong, and Red Tea from Blueberry Leaves. Food Sci. Nutr..

[142] Obadi M., Sun J., Xu B. (2021). Highland Barley: Chemical Composition, Bioactive Compounds, Health Effects, and Applications. Food Res. Int..

[143] Huang K., El-Seedi H.R., Xu B. (2022). Critical Review on Chemical Compositions and Health-Promoting Effects of Mushroom *Agaricus Blazei* Murill. Curr. Res. Food Sci..

[144] Pampoukis G., Zwietering M.H., den Besten H.M.W. (2024). Ranking Factors Affecting the Decontamination Efficacy of Non-Thermal Plasma: The Approach of Dissipated Power per Plasma Volume through Machine Learning Modeling. Innov. Food Sci. Emerg. Technol..

[145] Xiang Q., Liu X., Li J., Ding T., Zhang H., Zhang X., Bai Y. (2018). Influences of Cold Atmospheric Plasma on Microbial Safety, Physicochemical and Sensorial Qualities of Meat Products. J. Food Sci. Technol..

[146] Gavahian M., Chu Y.-H., Mousavi Khaneghah A., Barba F.J., Misra N.N. (2018). A Critical Analysis of the Cold Plasma Induced Lipid Oxidation in Foods. Trends Food Sci. Technol..

[147] Liang Y., Ma L., Xu Q., Tian X., Sun L., Cai J. (2025). Synergistic Treatment with Ozone Water and Morpholine Fatty Acid Salts Improves Postharvest Quality in Mandarin Oranges. Foods.

[148] Manzoor M.F., Shabbir U., Gilani S.M., Sameen A., Ahmad N., Siddique R., Ahmed Z., Qayyum A., Rehman A. (2022). Characterization of Bioactive Fatty Acids and Oxidative Stability of Microwave Vacuum Dried Fish Powder Supplemented Extruded Product. Food Sci. Technol..

[149] Zhou Y., Wu J., Monto A.R., Yuan L., Gao R. (2025). Elevated Levels of Branched Chain Fatty Acids in Low-Salt Fish Sauce by Co-Fermentation: Flavor Improvement and Metabolism Analysis. J. Sci. Food Agric..

[150] Zhang L., Li Q., Bao Y., Tan Y., Lametsch R., Hong H., Luo Y. (2024). Recent Advances on Characterization of Protein Oxidation in Aquatic Products: A Comprehensive Review. Crit. Rev. Food Sci. Nutr..

[151] Xu W., Bao Y., Zhou Y., Hong H., Gao R. (2024). Effect of Protein Oxidation on the Structure and Emulsifying Properties of Fish Gelatin. Food Res. Int..

[152] Liu Y., Mubango E., Dou P., Bao Y., Tan Y., Luo Y., Li X., Hong H. (2023). Insight into the Protein Oxidation Impact on the Surface Properties of Myofibrillar Proteins from Bighead Carp. Food Chem..

[153] Pan J., Li C., Liu X., He L., Zhang M., Huang S., Huang S., Liu Y., Zhang Y., Jin G. (2022). A Multivariate Insight into the Organoleptic Properties of Porcine Muscle by Ultrasound-Assisted Brining: Protein Oxidation, Water State and Microstructure. LWT.

[154] Tahir H.E., Xiaobo Z., Jianbo X., Mahunu G.K., Jiyong S., Xu J.-L., Sun D.-W. (2019). Recent Progress in Rapid Analyses of Vitamins, Phenolic, and Volatile Compounds in Foods Using Vibrational Spectroscopy Combined with Chemometrics: A Review. Food Anal. Methods.

[155] Liu F., Delchier N., Bao Y., Xie C., Li Y., Liu X., Yu X., Li L., Jin M., Yan J.-K. (2025). Chemical Oxidation of B Vitamins in Food Systems: Mechanisms, Matrix Effects, and Preservation Strategies. Compr. Rev. Food Sci. Food Saf..

[156] Zhang Z.-H., Chen J., Huang X., Aadil R.M., Li B., Gao X. (2024). Natural Pigments in the Food Industry: Enhancing Stability, Nutritional Benefits, and Gut Microbiome Health. Food Chem..

[157] Chamorro F., Carpena M., Fraga-Corral M., Echave J., Riaz Rajoka M.S., Barba F.J., Cao H., Xiao J., Prieto M.A., Simal-Gandara J. (2022). Valorization of Kiwi Agricultural Waste and Industry By-Products by Recovering Bioactive Compounds and Applications as Food Additives: A Circular Economy Model. Food Chem..

[158] Laroque D.A., Seó S.T., Valencia G.A., Laurindo J.B., Carciofi B.A.M. (2022). Cold Plasma in Food Processing: Design, Mechanisms, and Application. J. Food Eng..

[159] Umair M., Sultana T., Xun S., Jabbar S., Riaz Rajoka M.S., Albahi A., Abid M., Ranjha M.M.A.N., El-Seedi H.R., Xie F. (2023). Advances in the Application of Functional Nanomaterial and Cold Plasma for the Fresh-keeping Active Packaging of Meat. Food Sci. Nutr..

